# Comparison of Antimicrobial Activity of Chlorhexidine, Coconut Oil, Probiotics, and Ketoconazole on* Candida albicans* Isolated in Children with Early Childhood Caries: An In Vitro Study

**DOI:** 10.1155/2016/7061587

**Published:** 2016-03-14

**Authors:** Beena Shino, Faizal C. Peedikayil, Shyamala R. Jaiprakash, Gufran Ahmed Bijapur, Soni Kottayi, Deepak Jose

**Affiliations:** ^1^Department of Pedodontics, Kannur Dental College, Kerala 670612, India; ^2^Department of Microbiology, Kannur Medical College, Kerala 670612, India

## Abstract

*Background*. Early childhood caries (ECC) is associated with early colonisation and high levels of cariogenic microorganisms. With* C. albicans* being one of those, there is a need to determine the effectiveness of various chemotherapeutic agents against it. The study is aimed at isolating* Candida* species in children with ECC and at studying the antifungal effect of coconut oil, probiotics,* Lactobacillus*, and 0.2% chlorhexidine on* C. albicans* in comparison with ketoconazole.* Materials and Methods*. Samples were collected using sterile cotton swabs, swabbed on the tooth surfaces from children with ECC of 3 to 6 yrs and streaked on Sabouraud dextrose agar (HI Media) plates and incubated in a 5% CO_2_ enriched atmosphere at 37°C for 24 hours.* Candida* was isolated and its susceptibility to probiotics, chlorhexidine, ketoconazole, and coconut oil was determined using Disc Diffusion method.* Results*. The mean zone of inhibition for chlorhexidine was 21.8 mm, whereas for coconut oil it was 16.8 mm, for probiotics it was 13.5 mm, and for ketoconazole it was 22.3 mm. The difference between the groups was not statistically significant (Chi-square value 7.42, *P* value 0.06).* Conclusion*. Chlorhexidine and coconut oil have shown significant antifungal activity which is comparable with ketoconazole.

## 1. Introduction

The disease of early childhood caries (ECC) is defined as the presence of one or more decayed (noncavitated or cavitated lesions), missing (due to caries), or filled tooth surfaces in any primary tooth in a child 71 months of age or younger. In children younger than 3 years of age, any sign of smooth surface caries is indicative of severe early childhood caries (S-ECC) [1]. It is associated with early colonisation and high levels of cariogenic microorganism, high levels of dental plaque, enamel defects in the primary teeth, and childhood diets rich in sugar and carbohydrate. These primary risk factors interact to produce an acidic environment in the dental plaque causing decalcification of enamel and dentin.* Streptococcus mutans*,* Lactobacilli*, and* Candida albicans* are the predominant microorganisms found in dental plaque associated with a caries lesion [2]. Several antimicrobial agents have been introduced with the goal of suppressing the cariogenic bacteria, resulting in a remarkable reduction in* S*.* mutans* levels [3]. The contribution of* C. albicans* to overall microbial acid formation appears to be important, as it ferments glucose and maltose, producing both acid and gas [4]. The presence of* Candida* has been shown to enhance the adherence of* S. mutans* to the oral biofilm and carious tooth substance in vitro [5]. Research on the chemotherapeutic approaches to prevent or reduce the levels of* C. albicans* resulting in ECC has been limited, and there is a need to determine the effectiveness of various chemotherapeutic agents against* C. albicans* to reduce the caries experience in children.

Antifungal agents, such as azoles (fluconazole, ketoconazole) and polyenes (amphotericin B or nystatin), are commonly used to control the candidal infections [6]. Chlorhexidine has a wide spectrum of antimicrobial activity and is used as a topical therapeutic supplement. It is also capable of inhibiting the candidal adhesion to biological and inert surfaces [7]. Coconut (*Cocos nucifera*) is the unique source of various natural products, useful for the development of medicines against various diseases. The parts of its fruit like coconut kernel and tender coconut water are of a great medicinal value because of its antimicrobial and antioxidant property [8]. Probiotic bacteria have been used to modify microfloral ecosystems and have shown some success as a therapeutic for oral diseases [9].

This study aims to test the susceptibility of* C. albicans* isolates from children with ECC to the antifungal, ketoconazole, mouth rinse, chlorhexidine, coconut oil, and probiotic and to compare their antimicrobial efficacy.

## 2. Materials and Methods

Children with early childhood caries (ECC) in the age group of 3 to 6 yrs consulting the Outpatient Department of Pediatric And Preventive Dentistry, Kannur Dental College, Anjarakandy, are taken as subjects for the study. Children who were on topical or systemic antibiotics or antifungals are excluded from the study. Informed written consent was obtained from the accompanying parent/guardian of the child. The caries experience (dmfs index) of the children was recorded using visible light, mouth mirror, and CPI probe and based on the caries experience children with ECC were selected for the study.

**Figure 1:**
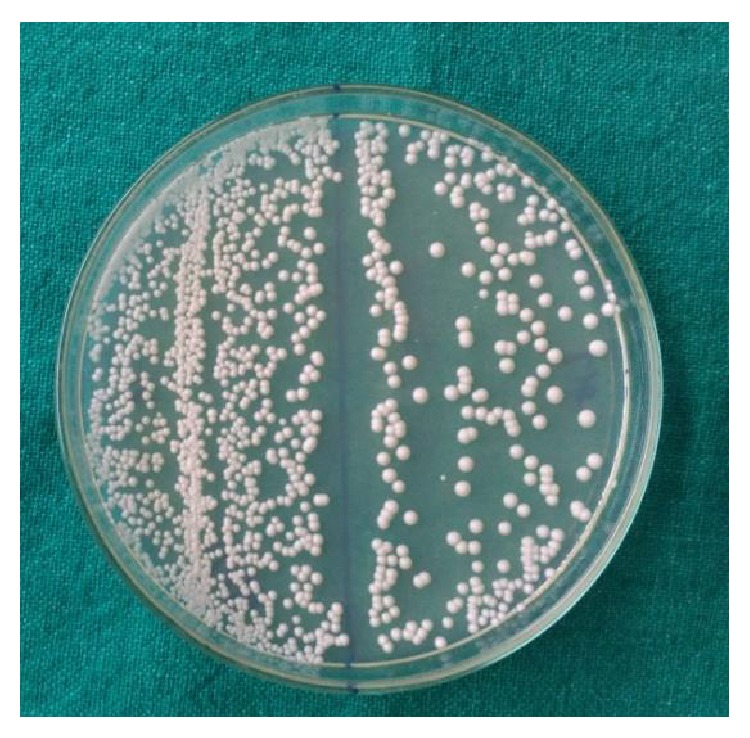
Candidal growth on SDA.

Samples were collected using sterile cotton swabs, swabbed over the buccal, lingual, proximal, and cervical portion of the teeth, and were immediately transferred to the lab for microbiological analysis. The samples were streaked onto Sabouraud dextrose agar (HI Media) plates supplemented with antibiotics (chloramphenicol 50 *μ*g/dL) and incubated in a 5% CO_2_ enriched atmosphere at 37°C for 24 hours and left at room temperature for further 24 hours. Growth of* Candida* appeared as cream or white coloured, smooth, and pasty colonies (Figure 1). Culture is said to be negative if there is no growth even after 72 hours of incubation. Candidal species identification is done by Germ tube test and morphology on corn meal agar (Dalmau Plate Culture method) read after 48 hours. Twenty such positive samples of* C. albicans* were obtained.

Antifungal activity of 2% ketoconazole (Kevon^R^), 0.2% chlorhexidine (Hexidine mouthwash), probiotics (Vizylac^R^, lactic acid* Bacillus *120*∗*10^6^), and coconut oil against* C. albicans* is tested by Kirby Bauer's Disc Diffusion method. 0.2% chlorhexidine, coconut oil and probiotics (Vizylac, lactic acid* Bacillus*), and 2% ketoconazole were applied on filter paper discs of 6 mm separately (4.0 *μ*L/disc) and allowed to dry.

**Figure 2:**
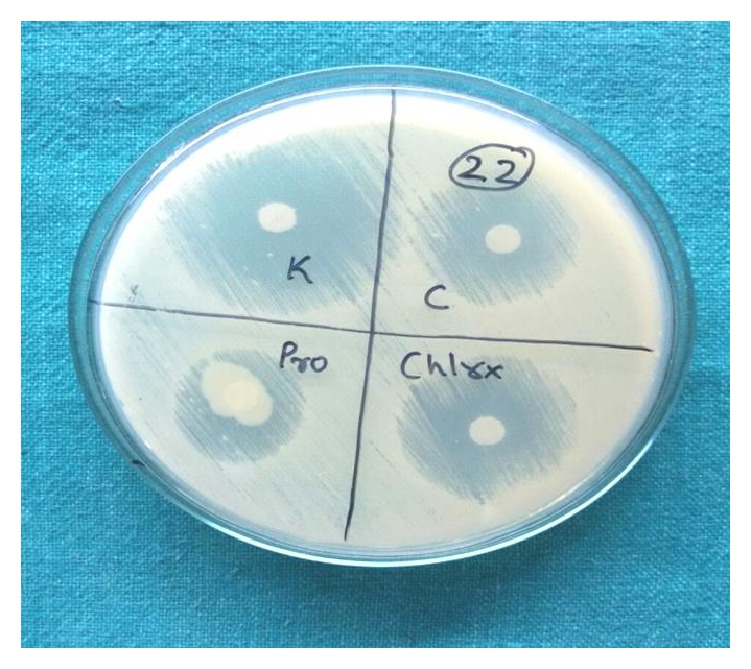
Zone of inhibition observed around the disc.

Suspensions of* C. albicans* were prepared in saline solution adjusted to the turbidity of 0.5 McFarland and streaked onto Mueller-Hinton agar supplemented with 1% glucose evenly. Then the discs of chlorhexidine, coconut oil, probiotics, and ketoconazole are placed on its surface at equal distance. This was done for all the 20 isolates of* Candida*. Then the plates were incubated at 37°C for 24 hours and observed for the zone of inhibition around the disc (Figure 2), which will be measured and compared.

## 3. Results

*Candida* was identified by its morphological features of cream, smooth, pasty convex colonies on SDA. Germ tube formation and the formation of conidiospores confirmed the presence of* C. albicans*. The antifungal susceptibility test showed that* C. albicans* was susceptible to ketoconazole, chlorhexidine, coconut oil, and probiotics by having a clear zone of inhibition.

### 3.1. Data Analysis

The phenotypes and the susceptibility of the isolates to the antifungals were compared against one another by the nonparametric Kruskal-Wallis, for multiple independent groups, or Mann-Whitney, for two independent groups, tests.

**Figure 3:**
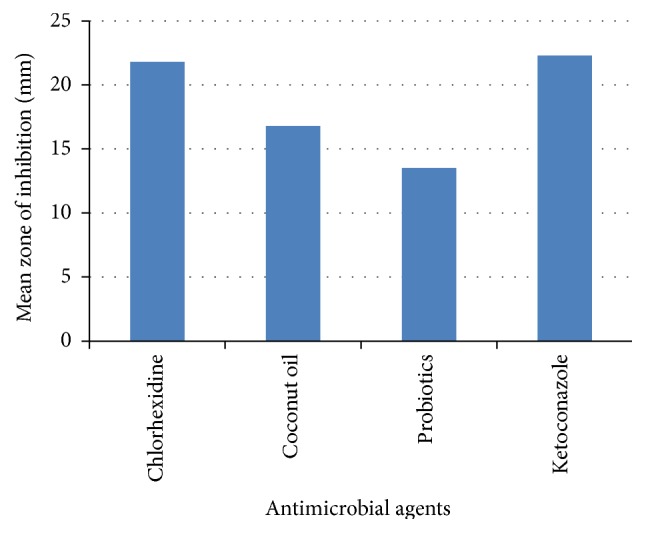
Mean of zone of inhibition of the antimicrobial agents against* Candida albicans*.

Table 1 shows comparison of zone of inhibition between different groups. It was found that the mean zone of inhibition for chlorhexidine was 21.8 mm, whereas for coconut oil it was 16.8 mm, for probiotics it was 13.5 mm, and for ketoconazole it was 22.3 mm (Figure 3). The difference between the groups was not statistically significant (Chi-square value 7.42, *P* value 0.06).

The zone of inhibition between CHX and ketoconazole is compared in Table 2. It was found that the mean zone of inhibition for chlorhexidine was 21.8 mm, whereas for ketoconazole it was 22.3 mm. The difference between the groups was not statistically significant (*P* value 0.54). Comparison of zone of inhibition between coconut oil and ketoconazole is shown in Table 3. The results show that the mean zone of inhibition for coconut oil was 12.8 mm, whereas for ketoconazole it was 22.3 mm. The difference between the groups was not statistically significant (*P* value 0.07). The zone of inhibition of probiotics and ketoconazole is shown in Table 4. A variation could be observed here as it shows that the mean zone of inhibition for probiotics was 13.5 mm whereas for ketoconazole it was 22.3 m and the difference between the groups was statistically significant (*P* value 0.02).

## 4. Discussion

The high incidence of sweetened substances in the diet of the children accompanied by poor oral hygiene, presence of caries lesions, and prolonged use of feeding bottles and pacifiers are the major factors related to the high prevalence of* C. albicans* in children. The highest colonisation site for* C. albicans* is provided by the carious lesion because it provides an ecologic niche for this microorganism [10].

Ketoconazole is an antifungal imidazole compound that has a significant activity against a broad range of superficial and systemic infections caused by pathogenic yeasts, dermatophytes, and filamentous fungi, including* C. albicans*. It stimulates phagocytosis and inhibits the filamentous growth of* C. albicans* [11]. The primary site of action of ketoconazole is shown as the inhibition of* C. albicans* respiration by inhibiting the activity of NADH oxidase at the mitochondrial level [12]. It causes direct membrane damage to* C. albicans* cells and inhibition of ergosterol biosynthesis, which is a characteristic constituent of yeast cell membranes. Hence, in the present study, ketoconazole is taken as the standard antifungal agent, against which other antimicrobial agents are tested.

Chlorhexidine digluconate is used as a broad spectrum antiseptic often incorporated in mouth rinses. It has a broad spectrum of activity against a variety of organisms, including* C. albicans* [13]. Biofilms on oral surfaces (dental plaque) are responsible for caries and periodontal disease. Chlorhexidine is capable of inhibiting candidal adhesion to biological and inert surfaces [14]. It acts as a fungicide and has a fungistatic function, leading to the coagulation of nucleoproteins and changes in cell walls allowing the possible escape of cytoplasmic components through the plasmalemma [15]. In the present study chlorhexidine showed significant antimicrobial activity against* C. albicans* with a mean zone of inhibition of 21.8 mm, and the difference with that of ketoconazole was not statistically significant (*P* value 0.54). In a similar study by Machado et al. [13] chlorhexidine solutions showed a reduction in the colony forming units of* Candida* biofilm.

Coconut oil is known to exhibit antimicrobial activity against* S. mutans* and* C. albicans* [16]. It has a unique role in the diet as an important physically functional food and is composed of medium chain fatty acids. It contains 92% saturated fatty acids, approximately 50% of which is lauric acid [17]. Monolaurin and other medium chain monoglycerides are shown to have the capacity to alter microbial cell walls, penetrate and disrupt cell membranes, and inhibit enzymes involved in energy production and nutrient transfer, leading to the death of the bacteria [18]. Bergsson et al. showed the susceptibility of* Candida albicans* to several fatty acids and their 1-monoglycerides [19]. In the present study coconut oil has shown antifungal activity that is comparable to that of ketoconazole.* C. albicans* was found to be highly susceptible to coconut oil in a similar study by Ogbolu et al. [20]. Coconut oil is also known to cause a significant reduction in gingivitis which can be attributed to decreased plaque accumulation and the anti-inflammatory, emollient effect of coconut oil [21].

According to a WHO/FAO report (2002), probiotics are “live micro-organisms which, when administered in adequate amounts, confer a health benefit on the host.” Use of probiotics and molecular genetics to replace and displace cariogenic bacteria with noncariogenic bacteria has shown promising results. Hatakka et al. [22] showed a reduced prevalence of* C. albicans* after taking probiotics in cheese. Results obtained by Kõll et al. [23] were markedly different, where it was reported that the growth of* C. albicans* was not inhibited by the probiotics. In the present study it has been found that probiotics inhibited the growth of* C. albicans*. However, it was lesser than that of chlorhexidine and coconut oil. The difference in the zone of inhibition with ketoconazole was statistically significant.

## 5. Conclusion

This study scientifically proves the antifungal activity of chlorhexidine, coconut oil, and probiotics. The antifungal activity of coconut oil is found to be higher than that of probiotics against* C. albicans*. Further studies must be carried out to determine the antimicrobial efficacy, the MIC, and MFC of these agents and more clinical studies have to be conducted to validate the same.

## Figures and Tables

**Table 1:** Comparison of zone of inhibition between different groups.

	*N*	Mean	Std. deviation	Chi-square	*P* value
Chlorhexidine	20	21.80	8.458	7.429	0.059_NS_
Coconut oil	20	16.80	12.846		
Probiotics	20	13.50	13.656		
Ketoconazole	20	22.30	15.076		
Total	80	18.60	13.033		

NS: not significant Kruskal-Wallis ANOVA.

**Table 2:** Comparison of zone of inhibition between CHX and ketoconazole.

	*N*	Mean	Std. deviation	Mean difference	*Z*-value	*P* value^*∗*^
Chlorhexidine	20	21.80	8.458	−0.5	−0.611	0.542
Ketoconazole	20	22.30	15.076			

^*∗*^Mann-Whitney *U* test.

**Table 3:** Comparison of zone of inhibition between coconut oil and ketoconazole.

	*N*	Mean	Std. deviation	Mean difference	*Z*-value	*P* value^*∗*^
Coconut oil	20	16.80	12.846	−5.5	−1.761	0.078
Ketoconazole	20	22.30	15.076			

^*∗*^Mann-Whitney *U* test.

**Table 4:** Comparison of zone of inhibition between probiotics and ketoconazole.

	*N*	Mean	Std. deviation	Mean difference	*Z*-value	*P* value^*∗*^
Probiotics	20	13.50	13.656	−8.8	−2.272	0.023
Ketoconazole	20	22.30	15.076			

^*∗*^Mann-Whitney *U* test.
